# Application of Machine Learning to Characterize the Permeate Quality in Pilot-Scale Vacuum-Assisted Air Gap Membrane Distillation Operation

**DOI:** 10.3390/membranes13110857

**Published:** 2023-10-26

**Authors:** Isabel Requena, Juan Antonio Andrés-Mañas, Juan Diego Gil, Guillermo Zaragoza

**Affiliations:** 1 CIEMAT-Plataforma Solar de Almería, Ctra. de Senés s/n, 04200 Tabernas, Spain; isabel.requena@psa.es (I.R.); guillermo.zaragoza@psa.es (G.Z.); 2 Centro Mixto CIESOL, ceia3, Universidad de Almería, Ctra. Sacramento s/n, 04120 Almería, Spain; juandiego.gil@ual.es

**Keywords:** membrane distillation, brine treatment, experimental, zero-liquid discharge, machine learning, modeling

## Abstract

Membrane distillation (MD) is a thermal desalination technique proposed for the valorization of residual brines that other operations such as reverse osmosis cannot treat. Previous studies have shown that vacuum-assisted air gap (V-AGMD) operation in commercial multi-envelope modules improves the performance of MD noticeably. However, the permeate quality at pilot scale has not been thoroughly characterized so far. The aim of this study is, therefore, to assess and model the effect of the main operating conditions (feed flow rate, inlet temperatures, and feed salinity) on the permeate quality. Results from different steady-state experiments allowed to estimate descriptive metrics such as the salt rejection factor (SRF) and the membrane leak ratio (MLR). Given their non-linear behavior, these metrics were subsequently modeled using artificial neural networks (ANN) to estimate the permeate quality in the whole scope of operating conditions. Acceptable SRF results with MLR values lower than 0.2% confirmed the validity of MD as an operation for the treatment of concentrated brines, although the salinity of the resulting permeate does not comply in all cases with that permitted for human consumption.

## 1. Introduction

Water scarcity is a major problem nowadays in both coastal and inland areas [1]. Desalination of sea and brackish water has become the most widespread solution to alleviate this issue [2]. Membrane technologies are the most used at industrial scale. Reverse osmosis (RO) is the most well-assessed technology for decades. Results obtained at both laboratory and industrial scale have allowed the development of new membranes and modules, and also numerous performance and costs models. All this experience throughout the time leads to RO being the most economically competitive sea- and brackish water desalination operation so far [3,4]. RO plants produce 70% of the total permeate worldwide, followed by thermal desalination operations such as multi-stage flash (MSF) and multi-effect distillation (MED) [5], with 16% and 7%, respectively. However, with the increasing freshwater demand worldwide, the disposal of concentrated brines to the sea or to wells could be an environmental problem with major relevance in the near future [6]. This fact brings to light the need of alternative desalination technologies able to deal with the concentrated brines that RO cannot handle because of the increased osmotic pressure [7,8]. Apart from producing more freshwater, a proper brine management will also give the opportunity of recovering valuable minerals from that residue and thus establish a circular economy scheme with zero liquid discharge (ZLD) as the final goal [9,10,11]. Among these novel brine concentration technologies, membrane distillation (MD) is proposed to supplement RO [9,12].

MD is a thermal desalination technique implemented at pilot scale for two decades and driven by the vapor pressure gradient between both sides of a hydrophobic microporous membrane, not by osmotic pressure [13]. This fact makes possible the treatment of brines that cannot be handled by RO due to their high salinity. However, the main issue reported in high-salinity MD is pore wetting induced by salt ions, which occurs when the hydrophobicity of the membrane pores (related to the diameter of the pores and the surface tension of the feed) is reduced, facilitating thus the pass of salts through the membrane and hence worsening the permeate quality [14]. Moreover, in the worst case, crystal growth can occur and cause severe irreversible membrane damage [11,15]. Membranes made of different materials and with different structures have been tested at a laboratory scale [16,17,18]. However, only a few studies have assessed the performance of MD at high salinity up to date. Several studies about the treatment of brines with concentrations up to 240 g L${}^{-1}$ and with membrane areas below 0.02 m${}^{2}$ have been published, considering the MD operational modes direct contact (DCMD) [19,20], air gap (AGMD) [21,22], and vacuum (VMD) [23]. A minor upscaling up to almost 0.2 m${}^{2}$ membrane area was later made. Valuable information about the treatment of real RO brine was provided in these studies carried out in DCMD [24] and VMD [25] operational modes. However, these results at a lab scale can hardly be extrapolated to bigger MD modules, therefore experimental evaluations in commercial-scale devices are mandatory to cover the current lack of knowledge [18].

One of the first assessments of pilot-scale MD modules in which permeate quality was considered in detail was published by Minier-Matar et al. [26]. Permeate electrical conductivity of <10 μS cm${}^{-1}$ was measured in the treatment of brine with concentration 70 g L${}^{-1}$ using two commercial plate-and-frame vacuum multi-effect MD (VMEMD) modules with membrane areas of 6.4 and 4.6 m${}^{2}$. This value is in the range of those obtained by Andrés-Mañas et al. [27] in a similar 6.4-m${}^{2}$ VMEMD module but operated with real Mediterranean seawater, which means a salt rejection factor (SRF) of above 99.98%. Regarding pilot-scale spiral-wound modules, values of permeate electrical conductivity up to 370 μS cm${}^{-1}$ (two orders of magnitude higher than those of lab-scale studies) were reported.

These results can be explained by the fact that the membrane pore diameter follows a Gaussian distribution, and hence as the membrane size increases, so does the number of pores with excessive size to maintain the hydrophobicity, increasing the liquid flow through the membrane. Winter et al. [28] evaluated a permeate gap (PGMD) single-envelope module with a membrane area of 10 m${}^{2}$ under feed salinities between 0 and 100 g L${}^{-1}$, but their work was not focused on permeate quality, although they presented values of permeate electrical conductivity. Soon after, Ruiz-Aguirre et al. [29] carried out a thorough analysis of the permeate electrical conductivity along the treatment time of a feed with 35 g L${}^{-1}$ marine salts in a similar PGMD module. The authors demonstrated that in the very beginning of the operation, the permeate itself acts rinsing the permeate channel, removing the liquid feed that has leaked through the membrane pores by microfiltration during the stand-by period, and therefore the permeate quality improved with time and stabilized below 20 μS cm${}^{-1}$. An alternative method to rinse the gap and maintain good permeate quality in a longer term was proposed by Schwantes et al. in a bench-scale single-envelope air-gap (AGMD) module [15]. Blowing air into the gap increased the absolute pressure on it, which is detrimental for vapor diffusion through the pores. The final result was a significant reduction of the permeate electrical conductivity when treating hypersaline feeds with up to 240 g kg${}^{-1}$ regarding the same tests performed without air sparging.

The subsequent development of multi-envelope AGMD modules increased the performance of the MD since higher feed flow rates with larger membrane area could be treated without an excessive hydraulic pressure drop inside the module [30]. This improvement in permeate flux and specific thermal consumption was characterized by Ruiz-Aguirre et al. [31]. Two multi-envelope modules named AS7 and AS24 were used, comprising 12 internal channels, but of different lengths (1.5 m in the former and 5 m in the latter). A multi-objective optimization of the trade-off between permeate flux and heat recovery was proposed for each one, but without taking into account the permeate quality.

Up to date, air suction from the gap and the membrane pores of multi-envelope AGMD modules has demonstrated to be the most successful solution to increase the MD performance. The vacuum-assisted air-gap MD (V-AGMD) operation is based on reducing the absolute pressure in the gap enough to improve vapor diffusion through the membrane pores, but without affecting the condensation inside the module. Values of permeate productivity reported in a module AS7 were up to 8.7 L h${}^{-1}$ m${}^{-2}$, similar to those obtained with the vacuum multi-effect technology (VMEMD). Besides, permeate electrical conductivity figures were in the same range (below 50 μS cm${}^{-1}$) [32], although the effect of vacuum on permeate quality must be thoroughly evaluated, especially in brine concentration processes. The only detailed study so far on this topic in pilot-scale modules was published by Ruiz-Aguirre et al. [33]. The authors showed that the SRF values of the modules AS7 and AS24 were worsened up to 2% by increasing the feed salinity up to 140 g L${}^{-1}$, and up to 1.5% when the absolute pressure in the gap was about 200 mbar, compared to experiments carried out in AGMD mode. On the contrary, the permeate quality in the single-envelope PGMD module remained almost unchanged and close to 100%. This suggests that the combined effect of high salinity and vacuum promotes membrane wetting. To quantify it, a performance parameter named membrane leak ratio (MLR) was introduced by the authors in the same study, which is defined as the ratio of feed that passes through the membrane pores in operation. In the worst case of SRF reported (97.2%), values of MLR < 0.12% were calculated, which brings to light the extreme dependence of permeate quality on the hydrophobicity of the membrane pores.

Subsequently, the performance of three V-AGMD modules AS7, AS24, and AS26 was compared, having the latter twice as many channels as the AS24 but with around half the length, which also reduces the circulation velocity by half, and thus the hydraulic pressure inside the channels. Experiments showed that the module AS26 outperformed the AS7 and the AS24, and provided the lowest specific thermal consumption reported to date in the MD literature: 40 kWh${}_{th}$ m${}^{-3}$, equivalent to GOR = 16.4. Therefore, the multi-envelope module AS26 is the strongest candidate to be part of a potential upscaled MD facility competitive with those of other technologies [34]. The authors provided SRF results higher than 98.2% and maximum membrane leak ratio (MLR) of 0.19% for several operating conditions, but no conclusive modeling of the effect of each variable on these quality parameters was performed on the module AS26.

Since permeate quality is subject to unknown defects on the membrane, the use of artificial neural networks is justified for modeling under different operating conditions [35,36]. Artificial Neural Networks (ANN) have emerged as a promising modeling tool in the realm of MD systems, especially for flux and thermal efficiency prediction [37] and fouling prediction [38,39]. One of the primary advantages of this methodology is its capability to effectively capture and fit almost all nonlinear processes. Furthermore, the inherent structure of the model enables retraining with additional experimental data, offering the potential for further enhancing prediction accuracy.

Multi-envelope modules operated in V-AGMD mode have been fully characterized due to their improved results. Concretely, the module AS26 has been modeled in depth in terms of heat recovery and permeate productivity, and operating conditions that optimize the performance have been established [40]. However, there are no studies focused specifically on assessing and modeling the permeate quality of the operation. This work presents for the first time the application of ANN to model and simulate the permeate quality of a pilot-scale MD system as a function of the operating conditions, providing a machine-learning framework for the application at hand. To do that, a comprehensive experimental campaign has been carried out at the solar MD facilities of CIEMAT-Plataforma Solar de Almería, using a pilot-scale multi-envelope module AS26 operated in V-AGMD mode. Outputs of the model are the key quality indicators salt rejection factor (SRF) and membrane leak ratio (MLR), whereas inputs are the operating conditions: evaporator channels inlet temperature (TEI), cooling channels inlet temperature (TCI), feed flow rate (FFR), and feed salinity (S). Finally, operating limits have been established, and techno-economical aspects of the operation in a wide range of conditions are discussed, focusing mainly on how both the permeate quality and the thermal efficiency are affected.

## 2. Materials and Methods

### 2.1. Materials: Experimental Facility

**Figure 1:**
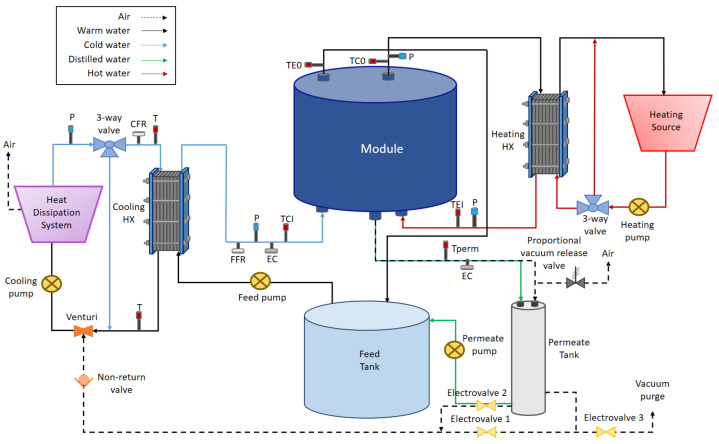
Layout of the MD system used in this work.

Experiments were carried out at the solar MD facilities of CIEMAT-Plataforma Solar de Almería (Spain). An MD unit manufactured by the Dutch company Aquastill was used. Figure 1 shows a scheme of the system which includes its main constituents.

The unit includes a multi-envelope spiral-wound module named AS26, with air gap configuration, and also provided by Aquastill. Table 1 shows its main features. It is internally designed alternating membrane sheets, condensation plates, and spacers, which delimit 12 evaporation channels, 12 cooling channels, and 24 permeate channels. Internal circulation of evaporation and cooling flows is countercurrent.

**Figure 2:**
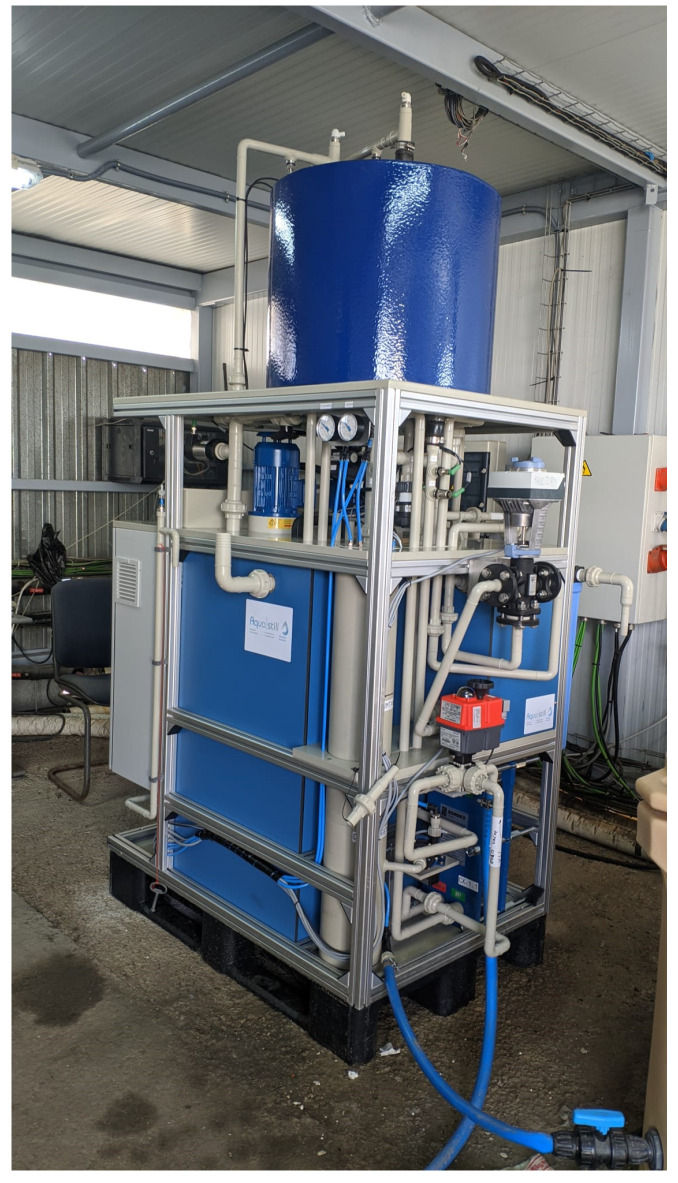
Commercial MD system used in the experimental campaign.

The module is integrated into the structure shown in Figure 2. It also bears two plate-and-frame heat exchangers, each one with a three-way valve that regulates the heating and cooling flows from external sources to maintain the two inlet temperatures around the desired setpoints. To measure the temperatures, four Pt-1000 sensors are installed at the inlet and outlet pipes of the module. The saline feed solution is contained in a 150-L tank and is pumped into the module through the inlet of the cooling channels. Its flow rate is measured and controlled by using a flowmeter, and its concentration is calculated from measurements of an electrical conductivity meter placed just before entering the module. Hydraulic pressure must be monitored to avoid surpassing the maximum recommended by the manufacturer (600 mbar in the case of the AS26 module). To do that, two pressure sensors are installed at the inlet of the evaporation and cooling channels. Vacuum is generated by Venturi effect using a narrowing tube inserted into the external cooling circuit, which sucks out air from the permeate tank and the gap of the module to the outside with no additional energy consumption than that of the cooling pump. Liquid permeate goes out of the module to a sealed vessel and is then intermittently discharged back to the feed tank to maintain the feed concentration. An electrical conductivity sensor in the permeate outlet allows monitoring the permeate quality. A system of solenoid valves allows discharging the permeate vessel without losing the vacuum inside the module. Finally, the data required to characterize the module are recorded by an *Esaware* PLC.

### 2.2. Methods

#### 2.2.1. Performance Metrics

In order to evaluate the permeate quality in MD, it is common in literature the use of the salt rejection factor (SRF), which represents the percentage of salts in the feed that are retained by the membrane:

(1) $$\mathrm{SRF}\;\left[\%\right]=100\cdot \left(\frac{S_{feed}-S_{permeate}}{S_{feed}}\right),$$

where $S_{feed}$ is the salinity of the feed solution and $S_{permeate}$ is the salinity of the permeate. To estimate these values, the electrical conductivity at 20 °C is measured, and conversion to salt concentration is then made using the correlation given in [33].

Pore diameter in polymeric membranes follows a Gaussian distribution around a mean value (0.32 μm in the case of the AS26 module). The pores with larger size will be less hydrophobic and, consequently, more prone to allow the pass of liquid saline feed to the permeate. Apart from salinity, as it will be thoroughly explained in Section 3.3, hydraulic pressure inside the module channels conditions the permeate quality critically. In this sense, the membrane leak ratio (MLR), initially described by Ruiz-Aguirre et al. [33]), takes into account both the feed flow rate (FFR) and the permeate flow rate (PFR), and is defined as the fraction of the liquid feed that passes through the membrane pores and contaminates the permeate (Equation (2)). Besides feed salinity, as this performance metric considers the hydraulics inside the module, it should be used to characterize the permeate quality in a more precise way than only using the SRF.

(2) $$\mathrm{MLR}\;\left[\%\right]=100\cdot \left(\frac{PFR}{FFR}\right)\left(\frac{S_{permeate}}{S_{feed}}\right)=100\cdot \left(\frac{PFR}{FFR}\right)\left(1-\frac{SRF}{100}\right).$$

#### 2.2.2. Experimental Procedure

The experimental steady-state tests carried out in the present campaign were maintained for at least 30 min, with 10–15 min of stand-by between them to ensure that operating conditions were stable after a change. Each experiment was replicated for a better uniformity of the results.

The feed in the tank is pumped through the cooling heat exchanger to cool it down up to the desired TCI by means of an external cooling source. Before entering the cooling channels of the module, the salinity of the feed is estimated using a Burkert in-line conductivity meter. This current is used for condensing the vapor inside the module and is separated from the evaporation channels by an air gap and a condensation sheet. The feed circulating along the cooling channels is preheated by the latent heat of condensation of the vapor passing through the membrane pores and, in a lesser extent, by the sensible heat of the permeate. After leaving the cooling channels, the feed is heated using external thermal energy up to the required temperature TEI, before re-entering the module through the evaporation channels. The resulting brine leaves the module and returns back to the feed tank, where it is mixed with the feed solution to maintain the steady state, avoiding temperature and concentration gradients inside them.

After condensation, the liquid permeate falls into a sealed two-chamber tank. It has a system of solenoid valves to close the upper chamber while the discharge is being made, so that the vacuum inside the module is not lost. Every time that 3.5 L of permeate are produced, the tank is emptied, being the activity of the permeate pump governed by the signals of a couple of level sensors. During the discharge, the electrical conductivity of the permeate is measured. To maintain the steady state, the permeate is also returned back to the feed tank.

Finally, performance metrics related to the permeate quality presented in Section 2.2.1 are calculated with the data recorded by the PLC. Table 2 summarises the ranges of the different input variables (TEI, FFR, TCI, and S) used in the experiments. Temperature ranges are the most commonly reported in pilot-scale MD literature, taking into account that TEI of 80 °C is the maximum limit established to avoid damaging the membrane structure, the spacers and the condensation sheets [41,42,43]. Moreover, this temperature is in the optimal range of operation with low-grade heat sources that can be coupled to MD, such as solar or waste heat [30,44]. FFR setpoints have been chosen to work at maximum of 400 mbar hydraulic pressure, far from the maximum limit allowed by the module.

#### 2.2.3. Artificial Neural Networks

**Figure 3:**
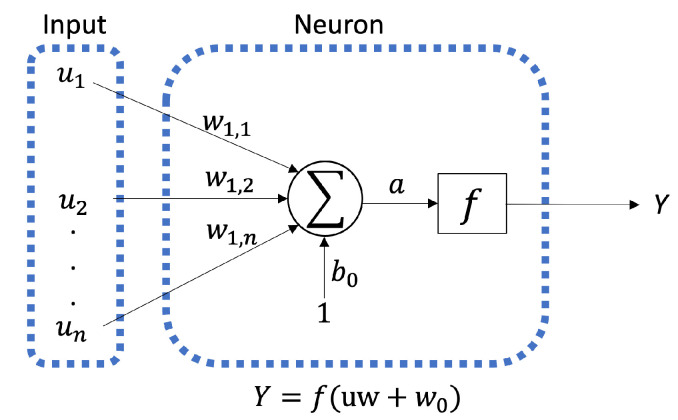
Schematic diagram of a neuron.

ANNs are mathematical operators composed of interconnected elements intended to process data when exposed to external stimuli. Their primary purpose is to mimic the functioning of biological neural networks [45]. The elements composing an ANNs, commonly referred to as neurons, demonstrate computational behavior similar to a processor, involving three essential operations, as depicted in Figure 3. These operations can be described as follows:

1. The inputs ($u_{1},u_{2},\ldots ,u_{n}$) undergo a multiplication process with the corresponding weights ($w_{1,1},w_{1,2},\ldots ,w_{1,n}$).
2. At the summing junction, the bias $b_{0}$ is added to the weighted inputs, resulting in:
  (3) $$a=u_{1}\cdot w_{1,1}+u_{2}\cdot w_{1,2}+\ldots +u_{m}\cdot w_{1,m}+b_{0}.$$
3. The value of *a* is transformed into a scalar output *Y* using a function *f*. This function can adopt different forms, including linear (Purelin) or sigmoidal (Logsig), among other possibilities. The computed outputs of neurons using these functions can be represented as follows:
  (4) Purelin:Y=f(a)=a,
  (5) $$\mathrm{Logsig}:Y=f\left(a\right)=\frac{1}{1+e^{a}}.$$

Neurons can be flexibly organized and combined in various ways, resulting in the topology or architecture of the ANN model. Typically, neurons are arranged into multiple layers, including input, hidden, and output layers. In the field of MD, one commonly used architecture is the multi-layer feed-forward perceptron (MLP) ANN [46]. In this type of ANN, the quantity of neurons in the input and output layers depends on the number of inputs and outputs in the system being studied. On the other hand, the designer has the flexibility to choose the number of hidden layers and the number of neurons within each hidden layer, as these parameters are adjustable.

After establishing the network architecture, the weights and biases are adjusted using a training algorithm. The back propagation (BP) algorithm is widely used for training MLP networks, as was detailed in [37]. The primary goal of this algorithm is to minimize a performance function by iteratively modifying the network’s weights and biases. The performance function used in this study is the root mean square error (RMSE), which is defined as follows:

(6) $$\mathrm{RMSE}=\sqrt{\frac{\sum_{i=1}^{M}\sum_{j=1}^{N}{\left(Y_{i,j}-{\hat{Y}}_{i,j}\right)}^{2}}{M\cdot N}},$$

where *M* represents the number of network outputs, *N* is the number of data points used for training, and Y${}_{i,j}$ and ${\hat{Y}}_{i,j}$ denote the experimental and predicted responses, respectively. Therefore, in each iteration, the BP algorithm adjusts the weights and biases in the direction that leads to a decrease in RMSE. The formula for one iteration of this algorithm is given by Demuth et al. (2014) [45] as:

(7) $$\lambda_{k+1}=\lambda_{k}-\delta \Delta_{k},$$

where $\lambda_{k}$ represents a vector containing the current network weights and biases, δ is the learning rate, $\Delta_{k}$ denotes the current gradient of the RMSE function, and *k* corresponds to the iteration number.

## 3. Results and Discussion

### 3.1. Experimental Results

Data registered by the MD unit were used to calculate the performance metrics SRF and MLR presented in Section 2.2.1. The reader can find in the Appendix A at the end of this manuscript the results obtained in the complete set of experiments.

### 3.2. Neural Network Model

#### 3.2.1. ANN Model Structure

The ANN-based model was developed using TEI, FFR, TCI, and S as inputs, while SRF and MLR were considered as outputs. The range considered for each variable was according to the setpoints used in the experimental campaign, see Table 2. The experimental data set was partitioned into three distinct subsets: (i) a training subset, comprising 70% of the samples, (ii) a validation subset, comprising 15% of the samples, and (iii) a test subset, consisting of 15% of the samples. In order to prevent overfitting problems through the training procedure, normalization of both the input and output model’s variables was conducted within the range of 0.1 to 0.9, using the normalization expression previously presented in [37,47]:

(8) $$Y_{n}=\left(1-B_{Low}-B_{Up}\right)\cdot \frac{Y_{k}-Y_{min}}{Y_{max}-Y_{min}}+B_{Up}.$$

In this expression, the parameter $Y_{n}$ stands for the normalized sample, $Y_{k}$ represents the real sample value, while $Y_{min}$ and $Y_{max}$ denote the minimum and maximum values of the variable to be normalized, respectively. Moreover, $B_{Up}$ and $B_{Low}$ are the lower and upper bounds of the normalized output, which, in this work, are fixed at $B_{Low}=B_{Up}=0.1$, according to the normalization range commented above.

**Figure 4:**
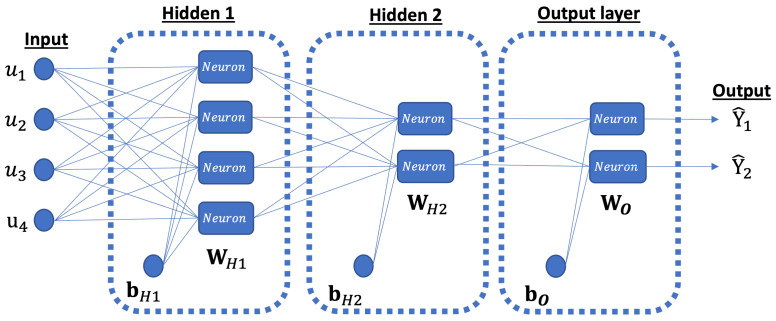
Schematic diagram of the obtained neural network. ${\hat{Y}}_{1}$ and ${\hat{Y}}_{1}$ are the SRF and MLR, respectively, while $u_{1}$, $u_{2}$, $u_{3}$, and $u_{4}$ are S, FFR, TCI, and TEI, respectively.

The training process of the ANN model was conducted through the Levenberg-Marquardt BP algorithm, available in the ANN Toolbox of MATLAB [48]. To find the optimal architecture, various ANN configurations were tested, varying the number of hidden layers from 1 to 2 and the number of neurons in each layer from 1 to 10. The function “Logsig” was applied in the hidden layer, while the “Purelin” function was employed in the output layer. The selection of the most appropriate architecture to characterize the MD module was based on the performance function (RMSE, see Equation (6)). The obtained optimal ANN model consists of four inputs, two hidden layers with four and two neurons, respectively, and an output layer with two neurons, as depicted in Figure 4.

**Figure 5:**
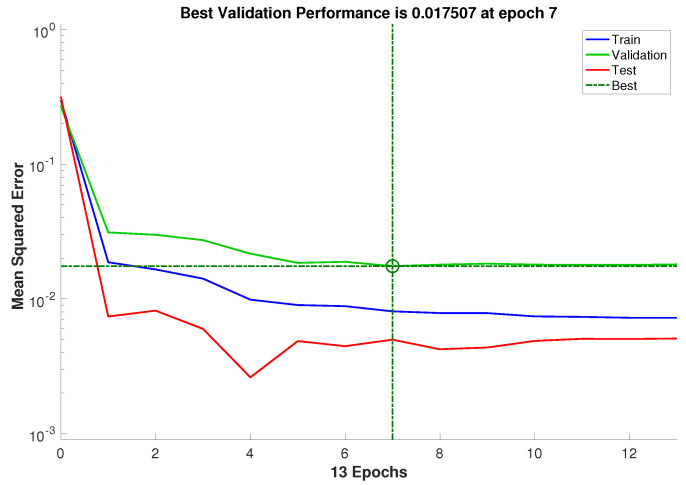
Evolution of the RMSE during the training procedure.

This specific feed-forward neural network architecture can be represented as MLP (4:2:2). Please note that the training process of this model was conducted iteratively, as mentioned in Section 2.2.3, until achieving a sufficiently small RMSE-value in the validation subset. For the optimal network, the training process was stopped after 7 iterations, resulting in an RMSE of 0.017 for the validation data subset, see Figure 5. The mathematical representation of the obtained ANN model can be expressed as follows:

(9) $$\hat{Y}=f_{O}\left(W_{O}\cdot f_{H2}\left(W_{H2}\cdot f_{H1}\left(W_{H1}\cdot u+b_{H1}\right)+b_{H2}\right)+b_{O}\right),$$

where $W_{H1}$ and $W_{H2}$ represent the matrices for hidden layer 1 and 2, respectively, whereas $b_{H1}$ and $b_{H2}$ are the corresponding bias vectors. $f_{H1}$ and $f_{H2}$ are the function of the hidden layers (“Logsig”). $W_{O}$, $b_{O}$, and $f_{O}$ are the weigh matrix, bias and function (“Purelin”) of the output layer. The values of all the weights and biases are presented in Table 3. Note that, in this representation, **u** is the vector of inputs of the model.

#### 3.2.2. ANN Model Prediction Performance

**Figure 6:**
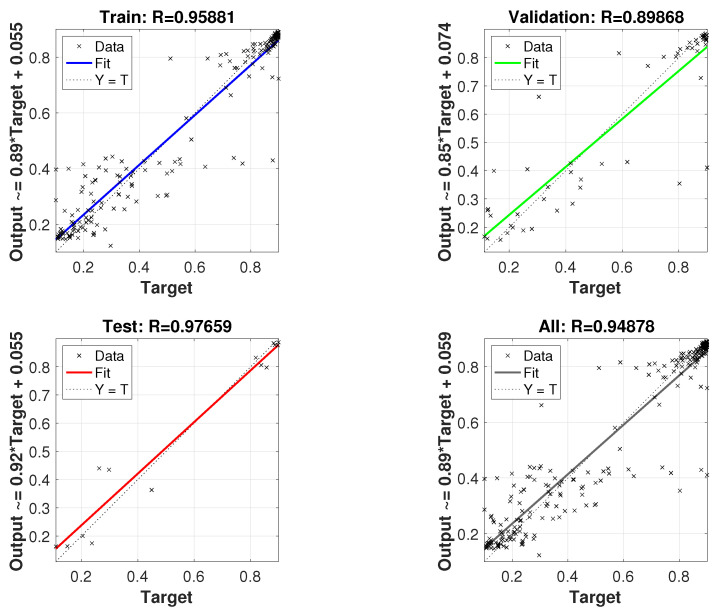
Validation of the model.

Figure 6 illustrates the agreement between the experimental data utilized during the training, validation, and test processes and the predicted values generated by the ANN model. As can be observed, the coefficient R is used as a performance indicator. In the case of the training subset, the value of coefficient R was higher than 0.95, the one of the validation tests was around 0.89, whereas the R-value of the test subset was 0.97. The closeness to one in all cases shows the good performance of the model. In particular, the value obtained in the test subset should be highlighted, since this subset includes data that has not been taken into account during the training process, and therefore data that are not known by the neural network.

**Figure 7:**
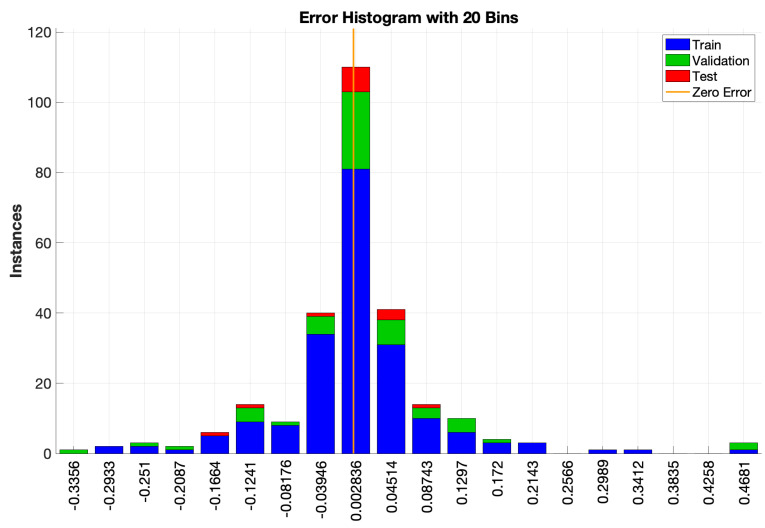
Error histogram.

Additionally, the error histogram of the training and test procedures is shown in Figure 7. As observed, the majority of the data used in the training and test procedures are concentrated around zero, with an error of around 0.002. On the contrary, the number of data concentrated on major errors is low, which again indicates that the model is capable of accurately estimating the behavior of the system.

### 3.3. Response Analysis

The neural network model developed in this work for the pilot-scale V-AGMD module AS26 allows to thoroughly assess the effect of the four inputs TEI, FFR, TCI, and S, on the outputs SRF and MLR.

**Figure 8:**
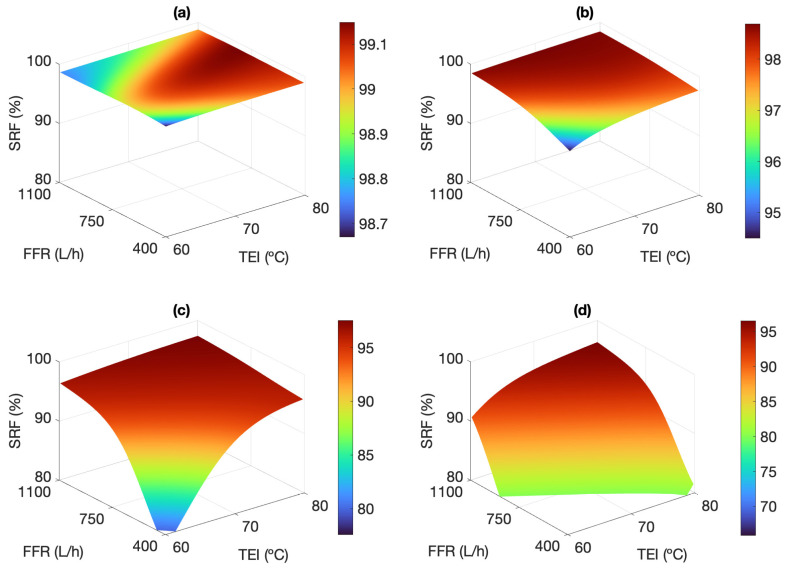
Response surfaces showing the influence of TEI and FFR on SRF, considering TCI = 25 °C, and S = 35.1 g L${}^{-1}$ (**a**), S = 105.2 g L${}^{-1}$ (**b**), S = 175.3 g L${}^{-1}$ (**c**), and S = 245.5 g L${}^{-1}$ (**d**). Experimental results below the x−y plane are missing because of unfeasible operating conditions.

Figure 8 shows the influence of TEI and FFR on the SRF for feed salinities between 35.1 and 245.5 g L${}^{-1}$. For each operating point, the SRF decreases with increasing salinity, since the leaked liquid is more saline. This affects negatively the hydrophobicity of the membrane pores, which act as microscopic water doors, therefore promoting the pass of feed as leak to the permeate [15]. Moreover, results bring to light a relationship between the SRF and the permeate productivity. Operation with high TEI improves not only the volume of permeate produced but also the separation performance. On the other hand, as salinity increases, the SRF has a more significant reduction the worse the operating conditions are to maintain the driving force. For TEI = 60 °C and FFR = 400 L h${}^{-1}$, the SRF is reduced from 99.92% at 35.1 g L${}^{-1}$ to even below 80% when the salinity is equal to or higher than 175.3 g L${}^{-1}$. This is equivalent to a permeate quality loss of 58.9 μS cm${}^{-1}$ to more than 58.6 mS cm${}^{-1}$, respectively. When the driving force improves with salinities up to 175.3 g L${}^{-1}$, TEI has no noticeable influence on the SRF if the FFR is maintained above 750 L h${}^{-1}$. Likewise, within that salinity range, the SRF gets almost independent of TEI when the FFR is maximum (1100 L h${}^{-1}$).

**Figure 9:**
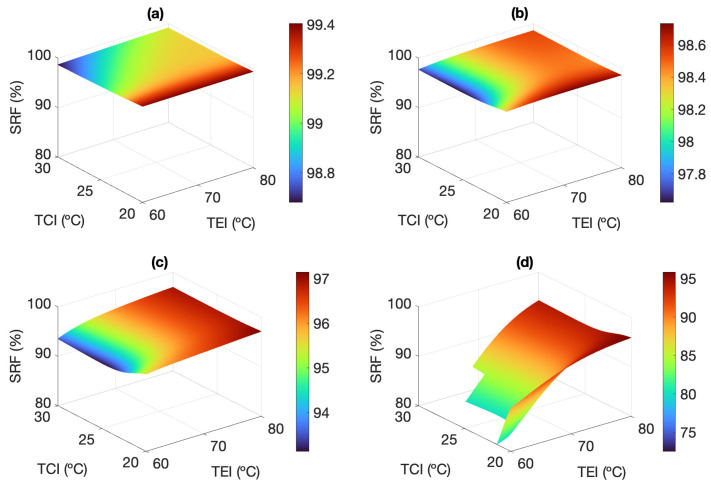
Response surfaces showing the influence of TEI and TCI on SRF, considering FFR = 750 L h${}^{-1}$ and S = 35.1 g L${}^{-1}$ (**a**), S = 105.2 g L${}^{-1}$ (**b**), S = 175.3 g L${}^{-1}$ (**c**), and S = 245.5 g L${}^{-1}$ (**d**). Experimental results below the x−y plane, and those of holes on the surfaces, are missing because of unfeasible operating conditions.

The observation that SRF is related to permeate productivity, i.e., to driving force, is confirmed by assessing the influence of TCI experimentally. TCI is the variable that affects the driving force the least because temperature changes within the range of 20–30 °C cause little variation in the vapor pressure of the feed, according to Antoine’s law [40]. The effect of TCI on the SRF is, therefore, small, as shown in Figure 9. Overall, for every salinity, lowering the TCI favors the vapor condensation and thus increases the permeate productivity, which leads to a slight increase of the SRF, but not higher than 1% in any case.

In the operation of commercial V-AGMD modules such as that studied in this work, the permeate quality is not only determined by the feed salinity but also by the internal design of the module and the membrane features. SRF is a useful parameter to measure the separation performance in an MD module, but not sufficient for a complete characterization of it. For a better quantification of permeate quality, the MLR takes into account the salinities of both currents and their flow rates.

**Figure 10:**
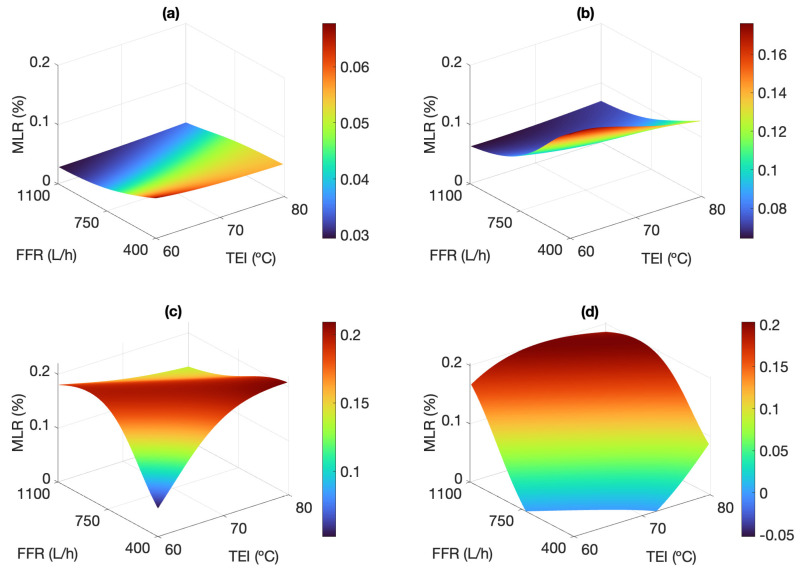
Response surfaces showing the influence of TEI and FFR on MLR, considering TCI = 25 °C, and S = 35.1 g L${}^{-1}$ (**a**), S = 105.2 g L${}^{-1}$ (**b**), S = 175.3 g L${}^{-1}$ (**c**), and S = 245.5 g L${}^{-1}$ (**d**). Experimental results below the x−y plane are missing because of unfeasible operating conditions.

The pore size of commercial polymeric membranes follows a Gaussian distribution, which implies the presence of pores with larger diameter than the average, commonly named pinholes. These hardly maintain the liquid-vapor boundary layer within them and have a greater propensity to let saline feed pass into the permeate. In an initial approach, the presence of these pinholes should result in constant leakage. However, in practice, the leaked feed is diluted by the pure permeate produced (with total absence of salts), all the more so the higher the permeate productivity is. Accordingly, the operating conditions also influence the product quality. Figure 10a,b clearly show this dilution effect: for salinities up to 105.2 g L${}^{-1}$, as FFR and TEI increase (thus favoring the driving force of the process) the estimated MLR values decrease up to 0.04% compared to low flow rate operation. It is also observed that at high FFR, the MLR remains constant at 0.035% and 0.06%, for feed salinities of 35.1 and 105.2 g L${}^{-1}$, respectively. According to Figure 8, these correspond to the highest SRFs, higher than 99.2%.

When estimating the permeate quality in V-AGMD operation, besides the dilution related to permeate flux, the hydraulic pressure and the vacuum level applied must also be considered. Their effect on the membrane performance can be significant. As a matter of fact, it has been experimentally observed that a higher SRF is not necessarily related to a lower MLR, since the latter also depends on the ratio between the permeate and feed flow rates (see Equation (2)). Inherently, a larger salt load promotes the pass of liquid feed through the pores with reduced hydrophobicity. A high hydraulic pressure drop into the internal channels of the module, corresponding to high feed flow rates, increases the inflow of thermal energy and therefore the permeate productivity, but also fosters the formation of those microscopic water doors. Figure 10 shows the opposing effects of hydraulic pressure and leaked feed dilution. For salinities up to 105.2 g L${}^{-1}$ (Figure 10a,b), the detrimental effect of hydraulic pressure at high flow rates is masked by permeate productivity, and thus the leaked feed is diluted by the pure permeate produced, even with low TEI. As feed salinity increases up to 175.3 g L${}^{-1}$, a competitive effect between the two aforementioned phenomena is established (Figure 10c). When TEI is equal to or less than 70 °C, an increasing FFR leads to higher MLRs because the dilution of the permeate affects that parameter in lesser extent than the leakage through the water doors. Contrarily, for TEI = 80 °C, the experimental results show some improvement in the MLR due to dilution as the FFR is increased, although values are still 3 times higher than those obtained at the lower TEI and the lower FFR (0.15% and 0.05%, respectively).

Considering the highest salinity assessed (245.5 g L${}^{-1}$), Figure 10d shows that the permeate quality is mainly affected by the hydraulic pressure drop into the channels, since vapour flux is reduced by the low water activity at that concentration. The SRF is reduced to 80% when FFR and TEI are lowered, but the MLR is also reduced to 0.1% or less. Since the volume of vapor produced is small, there is practically no dilution of the leaked feed, which leads to a small production mainly due to microfiltration, and with low quality (i.e., very low SRF), but still maintaining a high feed retention in the membrane (i.e., very low MLR).

Furthermore, applying strong vacuum levels in the gap of the module causes a detrimental additional suction force, although its use is justified by the remarkable increase in the performance of the pilot-scale MD module assessed in this study, regarding conventional AGMD operation [34]. The whole experimental campaign has been carried out with an absolute pressure in the gap of around 200 mbar, which is low enough to improve the permeate productivity, but high enough not to affect vapor condensation [32].

It must be highlighted that the MLR values remain below 0.21% in all cases, which indicates that, in the worst case, only 0.21% of the circulating feed will contaminate the permeate. This provides an evidence of the excellent hydrophobicity of the membrane used, even in the most unfavorable operating conditions.

### 3.4. Permeate Quality in a Thermally-Optimized V-AGMD Operation

Results in this work have confirmed that pilot-scale MD is a suitable technology for the desalination of hypersaline water sources and the valorization of saline residues from other technologies such as RO, in the context of ZLD. However, the high energy consumption of the operation up to date hinders the mass production of modules and thus their commercialization at a large scale. In the vast majority of scenarios, energy-related costs mean the largest fraction of CAPEX and OPEX. Therefore, it is difficult from a techno-economical point of view to propose an optimal operation of the process with other operating conditions than those that lead to the maximum thermal recovery, unless a free or very low-cost source of thermal energy could be exploited.

Previous studies about batch V-AGMD operation for brine concentration demonstrated that a gradual increase in the feed flow rate in time, as salinity increases, is required to maintain a sufficient thermal inflow that allows the maximum vapor diffusion through the pores, and thus the maximum heat recovery [40,49]. Table 4 summarises the optimal feed flow rates that maximize the thermal efficiency of the operation with the AS26 module, for each salinity assessed.

Experimental results of this campaign have brought to light a clear influence of the operating conditions on the permeate quality in the AS26 module (see Figure 8 and Figure 10). However, the effects were conflicting: both working at a reduced feed flow rate at low salinity, and increasing it at high salinity are beneficial actions for thermal consumption, but detrimental to the integrity of the membrane, since the leak of saline feed through the membranes is fostered. As shown in Table 4, permeate salinity increased from 0.27 to 7.62 g L${}^{-1}$ within the feed salinity range studied, whereas SRF reduced at about 97% and MLR increased consequently up to almost 0.18%. It is important to highlight that when working at the optimal feed flow rate for salinity of 35 g L${}^{-1}$ (400 L h${}^{-1}$), the salt rejection achieved in V-AGMD outperforms that of RO, which is commonly below 98% [50]. Besides, that permeate fulfills the salinity value of 0.35 g L${}^{-1}$ established by the WHO for human drinking water [51]. This would give V-AGMD the chance of being exploited as a seawater desalination operation, as long as the specific cost of water could be reduced up to the current RO levels.

**Figure 11:**
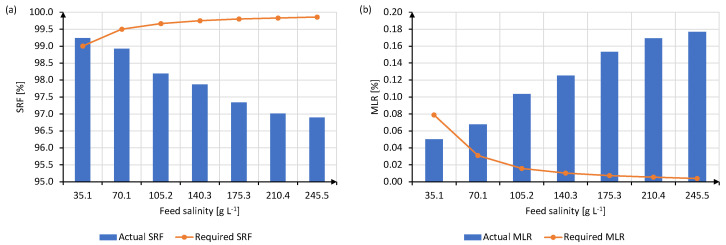
Values of (**a**) SRF and (**b**) MLR estimated for each feed salinity at the optimal operating conditions that maximize thermal efficiency, summarized in Table 4. Blue columns represent the actual values of each metric, whereas the orange dotted lines show the values they would have in each case considering the threshold of $S_{permeate}$ = 0.35 g L${}^{-1}$.

For the rest of feed salinities, this threshold is, however, surpassed. As depicted in Figure 11, from S = 70.1 g L${}^{-1}$ up, the required value of SRF (Figure 11a) corresponding to a permeate of 0.35 g L${}^{-1}$ is up to 3% higher than the actual experimental values estimated in each case. Similarly, actual values of MLR are even one order of magnitude higher than those necessary for satisfying the aforementioned requirement (Figure 11b), although with such low feed leak percentage (lower than 0.2%), the salt concentration in the most contaminated permeate does not exceed 8 g L${}^{-1}$. Therefore, besides its improved thermal efficiency, these figures make V-AGMD a suitable operation for the treatment of concentrated brines, even when operating conditions are not the most favorable to optimize the permeate quality.

## 4. Conclusions

V-AGMD operation on a commercial multi-envelope air gap module AS26 has been described in previous studies as the most energy efficient up to date. This makes MD technology a great candidate for the valorization of concentrates from other desalination operations that are less tolerant of high salinity, such as RO. However, no detailed studies about permeate quality in V-AGMD with this module and considering the feed salinity as a variable have been carried out so far. The present study is intended to fill the lack of information in the MD literature on this topic.

The experimental campaign comprised tests with different setpoints of inlet temperatures, feed salinity, and feed flow rate. With these results, two performance metrics that allow quantifying the quality of the permeate were calculated: SRF and MLR. The variables TEI, FFR, and S had a very noticeable influence on these two metrics, whereas TCI had a negligible effect.

Owing to the non-linearity of the experimental results, machine learning techniques were applied to characterize the two responses in relation to the aforementioned inputs, rather than common regression methods. Thus, the SRF and the MLR were modeled with an artificial neural network comprising two hidden layers with 4 and 2 perceptrons, respectively. Model validity was demonstrated by R-values close to one in the train (0.95), validation (0.89), and test (0.97) datasets.

Analysis of the experimental results showed two competing effects on permeate quality: the dilution of the feed leaked through the membrane pores and the hydraulic pressure. Under operating conditions that favor the driving force, i.e., TEI higher than 70 °C, FFR higher than 750 L h${}^{-1}$ and S up to 175.3 g L${}^{-1}$, their influence on permeate quality was little. Thus, the SRF values were within 99.99% with S = 35 g L${}^{-1}$ and 98.54% with S = 175.3 g L${}^{-1}$. These are equivalent to MLRs among 0.007% and 0.084%, respectively. In these cases, the vapor flux through the membrane pores is much larger than that of the feed leaked through the pinholes, so the dilution effect of the leak through the potential water doors allows maintaining high quality in the permeate. However, from an energy point of view, it must be taken into account that, in this salinity range, operating costs are optimized by reducing the feed flow rate.

When operating conditions hinder a high vapor diffusion through the membrane pores, the effect of hydraulic pressure surpasses that of leaked feed dilution. For feed salinity up to 70.1 g L${}^{-1}$, working with low TEI and FFR had a negative effect on both the SRF and the MLR, since the dilution effect diminished. However, at higher feed salinity, increasing productivity meant improving the SRF by up to 96%, but also worsening the MLR by up to 0.2%, since the reduced volume of permeate did not balance out the effect of the hydraulic pressure in the channels, together with the reduced hydrophobicity of the membrane due to the high salt load in the feed. Despite the aforementioned permeate quality values, no evidence of irreversible wetting or scaling was observed during the experimental campaign because the percentage of leaked feed in operation was very low.

This study has demonstrated the great tolerance to salinity of the pilot-scale V-AGMD module AS26 and the great hydrophobicity of the membranes currently used, which maintain their integrity even under conditions of high hydraulic pressure within the module channels, without signs of wetting or scaling. The main drawback identified was that the salinity requirement demanded by the WHO for drinking water (0.35 g L${}^{-1}$) was only met in the treatment of feed with marine concentration, although the separation of salts was very efficient.

In conclusion, considering both the thermal and the permeate quality performance, the V-AGMD module AS26 can be competitive for brine concentration. In any case, if the production of drinking water is seeked when using the modules for brine concentration, improved salt rejection by using superhydrophobic membranes must be considered in future works to maintain the permeate quality in a wider range of feed concentrations.

## Figures and Tables

**Table 1:** Characteristics of the MD module used in this study.

Feature	Value
Membrane area [m${}^{2}$]	25.92
Number of evaporation channels	12
Number of cooling channels	12
Channel length [m]	2.7
Mean pore diameter [µm]	0.32
Channel height [cm]	40
Channel width [mm]	2
Channel spacers porosity [%]	86.5
Air gap width [mm]	0.7–0.8
Air gap spacers porosity [%]	92.9
Membrane material	Low-density PE
Spacers material	PP
Condensation sheets thickness [μm]	80
Condensation sheets material	PET + Al

**Table 2:** List of setpoints used in the MD tests.

TEI${}_{\mathrm{sp}}$ [°C]	FFR${}_{\mathrm{sp}}$ [L h${}^{-1}$]	TCI${}_{\mathrm{sp}}$ [°C]	S${}_{\mathrm{sp}}$ [g L${}^{-1}$]
60	400	20	35.1
65	500	25	70.1
70	600	30	105.2
75	750		140.3
80	800		175.3
	900		210.4
	1100		245.5

**Table 3:** Optimal network weights and bias.

Weights and Biases of the ANN Model	
Input weight matrix	**W${}_{H1}$** = $\left|\begin{array}{cccc} 0.0899 & 0.5702 & -2.4336 & 0.1007\\ 1.4668 & -0.7317 & -0.0480 & -0.4964\\ 2.0210 & 0.0258 & -0.0138 & 0.1180\\ -1.5128 & -1.3960 & -0.7155 & 0.5511 \end{array}\right|$
Input bias vector	**b**${}_{H1}$ = $\left|\begin{array}{c} -2.2644\\ -0.9952\\ 0.5478\\ -1.3550 \end{array}\right|$
Hidden weight matrix	**W${}_{H2}$** = $\left|\begin{array}{cccc} 0.1747 & -2.0185 & -0.7157 & -0.1915\\ 0.7798 & -1.9531 & 0.7289 & 0.5311 \end{array}\right|$
Hidden bias vector	**b**${}_{H2}$ = $\left|\begin{array}{c} -1.5172\\ 1.9947 \end{array}\right|$
Output weight matrix	**W${}_{O}$** = $\left|\begin{array}{cc} 0.1073 & 1.2991\\ -0.4037 & 0.8154 \end{array}\right|$
Output bias vector	**b**${}_{O}$ = $\left|\begin{array}{c} -0.4125\\ -1.3375 \end{array}\right|$

**Table 4:** Values of permeate concentration, SRF, and MLR at the optimal FFR that maximizes the thermal efficiency. In all cases, TEI = 80 °C, TCI = 20 °C. Values of optimal FFR taken from [40]).

S${}_{\mathrm{feed}}$ [g L${}^{-1}$]	Optimal FFR [L h${}^{-1}$]	S${}_{\mathrm{permeate}}$ [g L${}^{-1}$]	Actual SRF [%]	Actual MLR [%]
35.1	400	0.27	99.2403	0.0502
70.1	400	0.76	98.9212	0.0679
105.2	421	1.90	98.1915	0.1035
140.3	639	2.98	97.8741	0.1252
175.3	840	4.65	97.3455	0.1531
210.4	1030	6.28	97.0145	0.1691
245.5	1100	7.62	96.8964	0.1768

## Data Availability

The data presented in this study are available on request from the corresponding author.

## Abbreviations

The following abbreviations are used in this manuscript:

AGMD	Air gap membrane distillation
ANN	Artificial neural network
AS7	Multi-envelope MD module with 7.2 m${}^{2}$ membrane area
AS24	Multi-envelope MD module with 24 m${}^{2}$ membrane area
AS26	Multi-envelope MD module with 25.92 m${}^{2}$ membrane area
BP	Back propagation
CAPEX	Capital expenditures [$]
DCMD	Direct contact membrane distillation
EC	Electrical conductivity [mS cm${}^{-1}$]
FFR	Feed flow rate [L h${}^{-1}$]
GOR	Gained output ratio
HX	Heat exchanger
MD	Membrane distillation
MED	Multi-effect distillation
MLP	Multi-layer feedforward perceptron
MLR	Membrane leak ratio [%]
MSF	Multi-stage flash
OPEX	Operational expenditures [$]
PFR	Permeate flow rate [L h${}^{-1}$]
PGMD	Permeate gap membrane distillation
PLC	Programmable logic controller
R	Regression coefficient
RMSE	Root mean square error
RO	Reverse osmosis
sp	Setpoint
S	Feed salinity [g L${}^{-1}$]
SRF	Salt rejection factor [%]
TCI	Cooling channels inlet temperature [°C]
TEI	Evaporation channels inlet temperature [°C]
VMD	Vacuum membrane distillation
V-AGMD	Vacuum-assisted air gap membrane distillation
VMEMD	Vacuum multi-effect membrane distillation
WHO	World Health Organization
ZLD	Zero-liquid discharge

## Appendix A

**Table A1:** Experimental results of SRF and MLR for different operating conditions.

TEI [°C]	FFR [L h${}^{-1}$]	TCI [°C]	S${}_{\mathrm{feed}}$ [g L${}^{-1}$]	SRF [%]	MLR [%]
60.0	1100	29.8	34.7	99.914	0.0310
70.0	400	25.1	35.0	99.699	0.0153
70.0	800	19.9	35.2	99.964	0.0210
59.7	400	20.0	35.2	99.894	0.0490
60.0	1100	20.0	35.3	99.950	0.0220
70.0	750	20.0	35.3	99.796	0.0117
80.0	400	25.0	35.4	99.896	0.0750
80.1	400	20.0	35.4	99.789	0.0750
79.8	800	20.0	35.5	99.979	0.0500
79.9	750	25.0	35.6	99.865	0.0900
70.0	1100	25.1	35.6	99.866	0.0680
60.0	750	24.9	35.7	99.704	0.0116
80.0	1100	20.1	35.8	99.977	0.0160
69.5	400	20.0	35.8	99.788	0.0127
80.0	800	25.0	35.9	99.969	0.0210
59.3	1100	25.4	36.0	99.984	0.0600
80.0	400	30.0	36.1	99.870	0.0830
70.0	750	30.0	36.1	99.758	0.0614
68.8	1100	21.3	36.1	99.986	0.0801
69.7	800	25.2	36.1	99.980	0.0118
59.3	800	20.0	36.2	99.981	0.0900
60.0	800	25.0	36.2	99.899	0.0424
80.0	1100	30.0	36.2	99.932	0.0435
60.0	400	25.2	36.2	99.819	0.0731
60.0	800	29.8	36.3	99.963	0.0342
69.0	1100	30.1	36.4	99.986	0.0708
79.6	1100	25.4	36.4	99.990	0.0745
79.0	800	30.0	36.4	99.979	0.0630
60.0	400	30.1	36.6	99.610	0.0726
69.0	400	30.8	36.9	99.884	0.0055
80.1	400	20.1	67.9	99.851	0.0960
60.0	600	19.9	68.3	99.776	0.0890
70.0	750	24.9	68.8	99.893	0.0524
70.0	600	30.0	68.8	99.867	0.0564
80.0	600	25.0	69.2	99.911	0.0575
80.0	750	20.0	69.3	99.878	0.0803
59.9	400	25.0	69.6	99.776	0.0683
69.8	400	20.1	69.7	99.794	0.0996
60.0	750	29.9	69.7	99.828	0.0506
59.8	1100	25.0	69.7	99.836	0.0598
79.7	400	25.1	70.3	99.809	0.0109
69.7	1100	20.4	70.4	99.919	0.0224
80.0	750	30.0	70.4	99.880	0.0195
60.0	750	19.9	70.5	99.704	0.0617
69.8	1100	30.0	70.5	99.902	0.0439
59.7	800	19.8	70.6	99.950	0.0206
79.6	1100	25.0	70.6	99.930	0.0432
69.8	400	30.1	70.7	99.754	0.0912
79.5	800	20.6	71.0	99.976	0.0161
79.1	400	29.9	71.2	99.899	0.0522
69.4	800	25.0	71.8	99.955	0.0223
59.9	800	29.5	72.0	99.936	0.0207
79.3	800	30.3	72.7	99.938	0.0358
60.0	750	19.8	104.2	99.650	0.0116
70.0	400	20.1	104.5	99.320	0.0236
70.0	750	25.0	104.6	99.552	0.0177
60.0	1100	24.9	104.9	99.649	0.0109
69.4	800	25.0	105.0	99.934	0.0271
79.9	400	25.0	105.0	99.440	0.0232
60.0	400	25.0	105.3	99.074	0.1539
69.9	1100	20.4	105.4	99.805	0.0937
80.0	800	20.8	105.4	99.795	0.0511
70.0	400	30.2	105.8	99.205	0.1376
60.0	800	29.9	105.8	99.910	0.0229
80.1	750	30.0	105.8	99.776	0.0110
80.2	750	20.0	105.9	99.830	0.0991
69.9	1100	30.0	105.9	99.748	0.0938
60.2	800	19.4	106.1	99.945	0.0209
60.0	750	30.0	106.3	99.304	0.0140
79.7	800	29.8	106.7	99.942	0.0292
80.0	1100	25.1	106.7	99.911	0.0509
70.0	600	20.2	139.5	99.023	0.0272
60.0	900	30.0	139.7	98.496	0.0201
60.0	1100	24.9	139.8	98.539	0.0311
70.0	750	24.9	139.8	98.902	0.0298
69.9	1100	20.3	140.1	99.370	0.0229
80.0	400	25.0	140.2	98.031	0.0455
80.0	1100	20.4	140.2	99.568	0.0209
60.0	750	30.0	140.5	96.735	0.0339
80.0	500	30.0	140.6	98.717	0.0296
80.0	750	20.0	140.6	99.236	0.0337
60.0	402	20.1	140.7	96.707	0.0363
60.0	400	25.0	140.7	90.657	0.0596
80.1	1100	25.0	140.7	99.451	0.0246
70.0	400	19.9	140.7	97.045	0.0542
75.0	600	20.0	140.9	99.165	0.0300
70.0	900	25.1	140.9	99.344	0.0193
70.0	400	30.0	140.9	93.901	0.1195
60.1	1100	29.8	141.0	98.663	0.0220
79.9	750	30.0	141.0	98.969	0.0340
60.0	750	20.1	141.1	98.257	0.0355
70.0	1100	29.9	141.3	99.019	0.0275
70.0	600	30.0	141.6	98.602	0.0229
80.1	800	30.0	142.1	99.451	0.0189
65.0	600	30.0	142.4	97.584	0.0270
65.0	800	29.9	142.5	98.574	0.0231
80.0	400	25.0	173.7	98.938	0.1521
60.0	750	20.1	173.8	99.018	0.1681
69.9	1100	20.8	174.3	99.670	0.1082
70.0	750	25.0	174.3	99.414	0.1322
80.0	750	20.0	174.8	99.721	0.1122
60.0	750	30.0	175.0	98.539	0.1842
60.0	1100	25.0	175.1	99.470	0.1908
70.0	400	20.0	175.5	98.585	0.1733
70.0	1100	29.9	175.8	99.661	0.1425
80.1	1100	25.1	176.7	99.808	0.1096
80.0	750	30.0	176.7	99.535	0.1215
60.0	750	30.0	209.9	95.080	0.1383
60.0	1100	25.0	210.3	98.113	0.1970
70.0	400	20.0	210.3	97.298	0.1201
80.0	400	25.0	210.6	97.036	0.1881
60.0	750	20.0	210.8	99.640	0.0398
70.0	1100	29.9	210.8	95.102	0.1678
80.0	750	20.0	210.8	98.360	0.1439
80.0	1100	25.3	211.1	99.385	0.1952
70.0	750	19.6	211.3	99.319	0.1394
80.0	750	29.9	211.7	98.260	0.1695
70.0	750	24.9	211.8	98.333	0.1215
70.2	1100	20.9	213.3	99.450	0.1404
80.0	400	20.0	234.9	99.992	0.1065
70.0	750	20.1	243.7	97.520	0.1160
60.0	750	25.0	245.1	87.449	0.0670
60.0	1100	20.4	245.2	97.248	0.1770
70.0	750	30.0	245.5	94.799	0.1329
80.0	750	25.0	245.7	97.650	0.1807
69.9	1100	25.2	247.4	95.999	0.1810
